# Acupotomy for knee osteoarthritis: A systematic review protocol

**DOI:** 10.1097/MD.0000000000017292

**Published:** 2019-09-27

**Authors:** Qinguang Xu, Hanjun Qiu, Zhixiong Zhu, Yueyi Wang, Shirong Yang, Zhuyi Si, Dong Shen, Xuezong Wang, Yi Di, Chaojie Lu, Xu Kang, Xiang Wang

**Affiliations:** a Department of Orthopedics and Traumatology, Fenghua Hospital of Traditional Chinese Medicine, Ningbo, Zhejiang Province; b Shi's Center of Orthopedics and Traumatology, Shuguang Hospital Affiliated to Shanghai University of Traditional Chinese Medicine, Shanghai, China.

**Keywords:** acupotomy, knee osteoarthritis, protocol, systematic review

## Abstract

**Background:**

Knee osteoarthritis (KOA) is the most common form of arthritis, leading to pain disability in seniors and increased health care utilization. Acupotomy has been widely used to treat KOA. But its efficiency has not been scientifically and methodically evaluated. The aim of this study is to evaluate the efficacy and safety of acupotomy for the treatment of patients with KOA.

**Methods:**

Relevant studies will be searched from the databases of PubMed, EMBASE, Cochrane Library, China Knowledge Resource Integrated Database, Weipu Database for Chinese Technical Periodicals, SinoMed, and Wanfang Database from their inception to June 10, 2019. Two researchers will independently select studies, collect data, and assess the methodology quality by the Cochrane risk of bias tool.

**Results:**

The systematic review will provide high-quality evidence to assess the efficacy and safety of acupotomy for KOA by pain, stiffness, and dysfunction of knee joint, and quality of life, as well as adverse events.

**Conclusion:**

The systematic review will provide evidence to assess the effectiveness and safety of acupotomy therapy for KOA patients.

**PROSPERO registration number:**

PROSPERO CRD42019132082

## 1 Introduction

Knee osteoarthritis (KOA) is one of the most common degenerative disease that causes disability in elderly people.^[1–3]^ An epidemiological study showed that about 30% of all adults have radiological signs of osteoarthritis (OA), 8.9% of the adults population has clinical significant OA of the knee or hip.^[4]^ And the probability of OA increases with age.^[5]^ For Chinese population, it has the similar trend. A study based on Chinese nationwide population suggested 8.1% total incidence rate of symptomatic KOA and increasing prevalence of KOA with age.^[6]^ In the rural regions of China, the prevalence is about 16.57%. For people over 70 years old, it is 29.25% for women and 24.71% for men.^[7]^ At present, for KOA patients, the aim of treatments mainly focus on relieving knee pain, enhancing mobility and function, as well as improving quality of life in KOA patients.^[8,9]^

Acupotomy is a new-style bladed needle which consists of flathead and cylindrical body. The instrument evolved from acupuncture needle.^[10]^ Acupotomy therapy is considered as a minimally invasive surgery that combined traditional Chinese acupuncture and modern surgical principle. So this therapy has been widely used clinically in traditional Chinese medicine hospital for many disease, applied to orthopedics and pain department.^[11]^ And the efficacy of acupotomy for KOA has been confirmed in animal experiment.^[12–14]^ However, no systematic review has been performed to evaluate the effectiveness and safety of acupotomy for KOA.

Therefore, this systematic review will assess the efficacy and safety of acupotomy for KOA. In this study, evidence-based medicine will be used to analyze and evaluate clinical randomized controlled trials (RCTs) in patients with KOA.

## 2 Methods

This systematic review protocol has been registered on PROSPERO (http://www.crd.york.ac.uk/PROSPERO/display_record.php?ID=CRD42019132082). The registration number is CRD42019132082. This protocol was performed in accordance with the preferred reporting items for systematic reviews and meta-analysis protocol (PRISMA-P). Ethical approval is unnecessary because this is a literature-based study.

### 2.1 Inclusion criteria for study selection

#### 2.1.1 Types of studies

All RCTs of acupotomy therapy for KOA without publication status restriction or writing language. Non-RCTs, quasi-RCTs, uncontrolled trials, reviews, case-controlled studies, animal trials, and laboratory studies will be excluded.

#### 2.1.2 Types of patients

KOA patients with definite diagnosis will be included. There will also be no limitations related to age, sex, disease duration, and disease severity.

#### 2.1.3 Types of interventions

The treatment group will be treated with acupotomy (there is no limit on the needle materials, treatment methods, and course of treatment). Because there is no false acupotomy reported in the literature and acupotomy commonly used in the acupuncture-moxibustion department. The control group will adopt the internationally recognized therapy such as block therapy or no treatment, acupuncture will also be included. Acupotomy with another active therapy versus the same therapy alone will also be investigated. Studies comparing 2 different types of acupotomy or surgical procedures will be expelled.

#### 2.1.4 Types of outcome measures

##### 2.1.4.1 Primary outcome

The primary outcome is Western Ontario and McMaster Universities Osteoarthritis Index (WOMAC). WOMAC is a self-report questionnaire for OA of the hip or knee, with higher scores indicating more serious pain, poorer physical function, and increased stiffness. It has been widely used as a tool by clinical investigators to assess patients with KOA.

##### 2.1.4.2 Secondary outcomes

Lequesne index and Medical Outcomes Study Short Form 36 health survey will be accepted as the secondary outcomes.

##### 2.1.4.3 Safety outcomes

The incidence and severity of side effects will be used to evaluate the safety. Any unexpected events occurred will be recorded.

### 2.2 Search methods for the identification of studies

#### 2.2.1 Electronic searches

Relevant studies will be searched in the following electronic databases from their inception to June 10, 2019: PubMed, EMBASE, Cochrane Library, China Knowledge Resource Integrated Database, Weipu Database for Chinese Technical Periodicals, SinoMed, and Wanfang Database. The search terms include KOA, gonarthrosis, osteoarthrosis, osteoarthropathy, arthralgia, small needle knife, acupotomy, needle knife, and RCTs. The equivalent search words will be used in the Chinese databases. The detailed strategies for searching the PubMed database will be presented in Table 1.

**Table 1:**
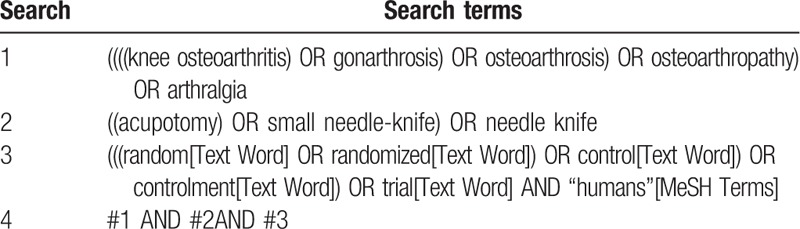
Search strategy used in PubMed.

#### 2.2.2 Searching other resources

Additionally, the international clinical trials registry platform, dissertation, and gray literature will also be searched to identify systematic reviews related to acupotomy for KOA. The relevant conference papers, journals will be retrieved manually.

### 2.3 Data collection and analysis

#### 2.3.1 Selection of studies

Two researchers independently conducted the processes. All literature will be imported to the endnote X9. And the duplicated data will be eliminated. We will screen the records after duplicates removed by 2 steps:

(1) reading the title and abstract,
(2) reading the full texts.

**Figure 1:**
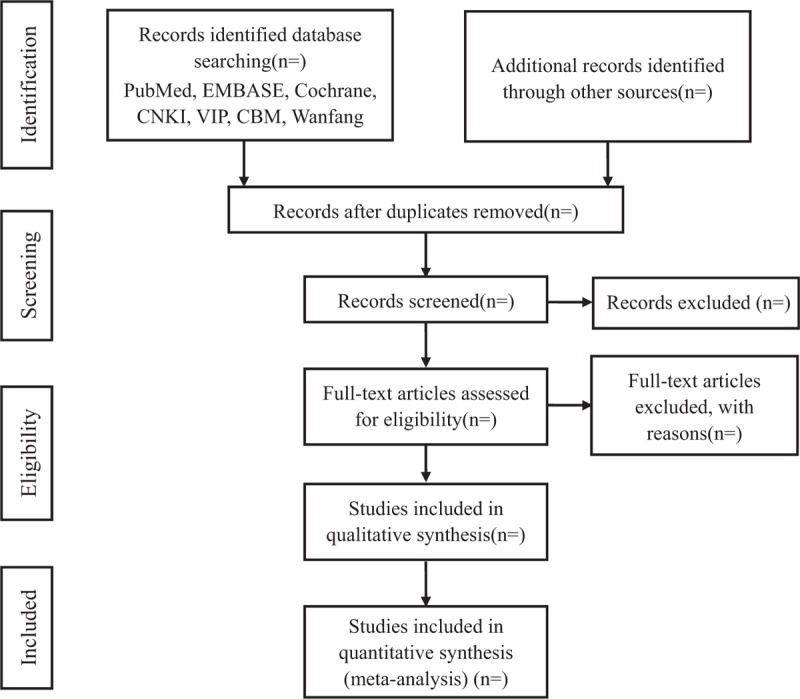
The PRISMA flow diagram of study selection process. PRISMA = preferred reporting items for systematic review and meta-analysis.

Whether a study will be included depended on the predefined criteria. Any study excluded should be labeled on full article. Any disagreement between 2 authors should reach consent by discussing with the third author. The process of study selection is summarized in a PRISMA flow diagram (Fig. 1).

#### 2.3.2 Data extraction and management

Before data extraction, a standard form will be prepared for data collection. Two researchers will independently extract data of the included studies and write on the form. Any disagreement will be solved by consensus. The following data will be extracted: the first author, publication year, participants characteristics, interventions, duration of treatment, follow-up, outcome assessment, research results, adverse events, and other detail information. We will contact the original author for complete information when necessary.

#### 2.3.3 Assessment of risk of bias

Two researchers will assess the risk of bias of included studies independently according to the Cochrane collaboration's tool.^[15]^ The tool comprise 7 aspects which are random sequence generation, allocation concealment, the blinding method for patients, researchers and outcomes assessors, incomplete outcome data, and selective reports. Every risk of bias will be classified as low, unclear, and high.^[16]^

#### 2.3.4 Measures of treatment effect

For continuous data, a mean difference or standardized mean difference with 95% confidence intervals will be applied. For dichotomous outcome data, the risk ratio with 95% confidence intervals will be used to evaluate the treatment effect.

#### 2.3.5 Missing data management

If the essential data are not provided, we will try to contact the corresponding author of the articles by email for complete data. If the missing data cannot be obtained, we will analyze the available data.

#### 2.3.6 Assessment of heterogeneity

The heterogeneity will be detected by the chi-squared test. When *I*^2^ is less than 50%, which indicate low heterogeneous. The heterogeneity is considered as fair. Otherwise, there is a significant heterogeneity among the included studies. Subgroup analysis will be performed to detect the potentials factors of heterogeneity.

#### 2.3.7 Subgroup analysis

If there is a significant heterogeneity in the included studies, subgroup analysis will be performed to detect the substantial heterogeneity based on the severity of KOA and types of acupotomy.

#### 2.3.8 Data synthesis

All data will be combined and analyzed by the Cochrane Collaboration software (Review Manager Version 5.2 for Windows; Copenhagen: The Nordic Cochrane Centre). When *I*^2^ value is less than 50% indicating low heterogeneous, the fixed-effect model will be applied to pool the data. Otherwise, a random-effect model will be used for data synthesis. In this situation, subgroup analysis will be also performed. After the subgroup analysis, if there still is obvious clinical heterogeneity, the data are inappropriate to be pooled, only descriptive analysis instead.

#### 2.3.9 Sensitivity analysis

When there are sufficient studies, sensitivity analysis will be performed to assess the robustness of studies according to methodological quality, sample size, and missing data.

#### 2.3.10 Reporting bias

Funnel plot will be performed when the included studies more than 10. Also, we will calculate the Egger regression and the Begger tests to check the asymmetry of funnel plot.

#### 2.3.11 Confidence in cumulative evidence

The quality of evidence will be assessed based on the grading of recommendations assessment, development, and evaluation system, include 4 levels: high, moderate, low, or very low.

## 3 Discussion

KOA is one of the most common orthopedic diseases, and its incidence rate has increased in recent years. At present, pharmacologic and non-pharmacologic approaches are the main choice for KOA. Numerous studies have reported that traditional Chinese can be used for KOA. And the acupotomy therapy is a miniature surgery with less pain.^[17]^ Although some studies results suggested that acupotomy can effectively improve the symptoms of KOA, its efficacy and safety have not been evaluated systematically.^[18–20]^ It is crucial to determine whether acupotomy is a good choice for KOA patients.

Therefore, the purpose of this proposed systematic review is to evaluate the efficacy and safety of acupotomy treatment in patients with KOA. We expect that our systematic review and meta-analysis will provide more evidence with deep understanding of acupotomy. There are some limitations in this review. Language and different measurements and tools may lead to the risk of heterogeneity.

## Author contributions

**Conceptualization:** Hanjun Qiu, Xiang Wang.

**Data curation:** Zhixiong Zhu, Xiang Wang.

**Formal analysis:** Hanjun Qiu, Zhuyi Si.

**Funding acquisition:** Yueyi Wang.

**Investigation:** Shirong Yang.

**Methodology:** Hanjun Qiu, Dong Shen, Xuezong Wang, Chaojie Lu, Xu Kang.

**Project administration:** Xiang Wang.

**Resources:** Zhuyi Si, Xiang Wang.

**Software:** Hanjun Qiu, Zhuyi Si, Yi Di.

**Supervision:** Hanjun Qiu, Xiang Wang.

**Validation:** Hanjun Qiu, Dong Shen, Yi Di, Xiang Wang.

**Visualization:** Dong Shen, Yi Di, Xu Kang, Xiang Wang.

**Writing – original draft:** Yi Di, Xiang Wang.

**Writing – review and editing:** Xu Kang, Xiang Wang.
